# Maxillomandibular Advancement with the Use of Virtual Surgical Planning and the CAD/CAM Technology in OSA Surgery: Volumetric Analysis of the Posterior Airway Space

**DOI:** 10.3390/medicina61020179

**Published:** 2025-01-22

**Authors:** Eleonora Segna, Funda Goker, Giulia Tirelli, Massimo Del Fabbro, Aldo Bruno Giannì, Giada Anna Beltramini, Diego Sergio Rossi

**Affiliations:** 1 Maxillo-Facial Surgery and Dental Unit, Fondazione IRCCS Ca’ Granda Ospedale Maggiore Policlinico, 20122 Milan, Italy; aldo.gianni@unimi.it (A.B.G.); giada.beltramini@unimi.it (G.A.B.); diego.rossi@policlinico.mi.it (D.S.R.); 2 Department of Biomedical, Surgical and Dental Sciences, University of Milano, 20122 Milan, Italy; funda.goker@unimi.it (F.G.); giulia.tirelli@unimi.it (G.T.)

**Keywords:** obstructive sleep apnea, orthognathic surgery, virtual planning, cone beam computed tomography, airway volume

## Abstract

*Background and Objectives*: Obstructive sleep apnea is an extremely diffuse pathology that, if left untreated, can lead to very serious cardiovascular consequences. The primary goal of treatment is to maintain airflow in the upper airway tract, which can be obtained thanks to orthognathic surgery such as maxillo-mandibular advancement (MMA). This procedure increases the volume of the posterior airway space (PAS)—a parameter considered fundamental in OSA physiology. However, the correlation between the degree of advancement, the volume increase, and the clinical improvement in OSA is not yet clear, even in patients who undergo virtual surgical planning. Aiming to test the correlation of these parameters and the role of PAS volume changes, we present our pre- and post-operative volumetric analysis of the PAS using cone beam computed tomography (CBCT) following CAD/CAM-assisted maxillomandibular advancement. *Materials and Methods*: We collected information from patients who underwent MMA for moderate or severe OSA, planned virtually with custom-made devices, between 2020 and 2022 at the Maxillofacial Surgery and Odontostomatology Unit of the Policlinico Hospital in Milan. The degree of mandibular advancement (pogonion antero-posterior advancement) was noted. All patients underwent pre- and post-operative CBCT and pre- and post-operative polysomnography to measure the Apnea–Hypopnea Index (AHI) parameters. Both exams were performed within six months before and after surgery. The surgeries were planned virtually along with the production of custom-made devices (cutting guides and mandibular osteosynthesis plates). Volumetric analysis of the PAS was performed pre- and post-CBCT images using medical segmentation software (Mimics, Materialise, Mimcs 26.0). *Results*: Ten patients (nine men and one woman) with a mean age of 51 years were included in this study. The mean pogonion advancement was 14.5 mm, ranging from 13.8 to 15.6. The mean pre-surgical AHI was 52.31 events/h, while the mean post-surgical AHI was 5.94 events/h (SD 5.34). The improvement in AHI was statistically significant (Wilcoxon matched-pairs signed-rank test, *p* value 0.004). The mean pre-surgical PAS volume was 8933 mm^3^, while the mean post-surgical volume was 10,609 mm^3^. In 8 out of 10 patients, the volume increased, with a mean increase of 2640 mm^3^ (max. 5183, min. 951), corresponding to a percentage increase variation ranging from 78% to 6%. In two patients, the volume decreased by 1591 (−16%) and 2767 mm^3^ (−31%), respectively. The difference between pre- and post-operative results was not statistically significant (paired *t*-test, *p* value 0.033). *Conclusions*: The results obtained confirm the efficacy of virtually planned MMA performed with custom-made devices in OSA therapy. However, they also show that PAS volume should not be used as a comprehensive parameter for OSA treatment evaluation because it does not always have a positive correlation with advancement and AHI.

## 1. Introduction

Obstructive sleep apnea (OSA) is a common public health problem, affecting just over 1 billion people worldwide. It is defined by the occurrence of a partial or complete collapse of the pharynx during sleep [1,2]. Like other sleep apnea syndromes (SAS), including central sleep apnea (CSA) and complex sleep apnea syndrome (CSAS), OSA cause sleeping hypoxemia, which can result in serious health consequences, primarily cardiovascular disease (CVD). In OSA, blood hypoxemia is a consequence of the total or partial collapse of the upper airway, inducing apnea and hypopnea. The symptoms of sleep apnea include loud snoring, episodes of breathing cessation, gasping, or choking during sleep, transient awakening, excessive daytime sleepiness, morning headaches, difficulty concentrating, irritability and mood changes, high blood pressure, nocturia, pain in the chest, and palpitations [3]. Some maxillofacial features can be correlated with OSA (maxillary hypoplasia, mandibular retrognathia, macroglossia, and glossoptosis). Nevertheless, its etiology could be multifactorial, with soft tissue thickness and the tonicity of the pharyngeal, laryngeal, and tongue muscles also playing a role. 

The diagnosis starts with clinical suspicion, supported by tools such as the Epworth Sleepiness Scale (ESS). It then requires both morphological and functional evaluations; Several radiographic methods enable craniofacial analysis and help in the diagnosis of OSA, including profile teleradiography, cone beam computed tomography (CBCT), computed tomography (CT), and magnetic resonance imaging (MRI) [4]. Fibroscopy of the upper airway and sleep endoscopy are also important steps in diagnosis and helping tailor the treatment. Additionally, polysomnography, along with the Apnea–Hypopnea Index (AHI)—defined as the total number of episodes of apnea and hypopnea per hour of sleep—is the main metric used to quantify the severity of sleep apnea (5–15, mild OSA; 15–30 moderate OSA; >30, severe OSA). Higher AHI values are associated with increased cardiovascular disease mortality and morbidity [5,6]. 

Besides daily behavior modifications (quitting smoking, stopping alcohol consumption, losing weight, and adjusting sleep posture) [7,8], continuous positive air pressure (C-PAP), which ensures high-pressure airflow, remains the first-choice conservative treatment despite difficulties in adherence and long-term tolerability [9,10]. Beyond C-PAP, many other interventions have been proposed to counter sleep obstruction and improve airflow in the upper airway [11]. These range from oral appliances, such as MADs (mandibular advancement devices), to hypoglossal nerve stimulators and surgical procedures aimed at increasing the volume of the upper airway by adjusting soft tissues (e.g., uvulo- and palatoplasty) or the position of bone structures. Rarely, tracheostomy may be needed to bypass the obstruction entirely. Maxillomandibular advancement (MMA), an orthognathic surgery that ensures the preservation of morphology and dental occlusion, offers a valuable choice for OSA correction, ensuring good and stable long-term results [12]. Moreover, the predictability and accuracy of this surgical technique have been improved thanks to CAD/CAM surgery [13].

There is no scientific consensus on the efficacy of OSA treatments, but it is globally accepted that the therapeutic target should be an AHI reduction of 50% or a decrease below 5. MMA is considered to achieve this goal by increasing the posterior airway space (PAS)—a predictor of airflow improvement [14]. A positive direct correlation between the two parameters is frequently reported [15], with PAS volume being directly linked with the degree of antero-posterior bimaxillary advancement and inversely linked with AHI reduction. This topic has not yet been analyzed in virtually planned MMA. Previous publications on OSA patients who underwent virtually planned orthognathic surgery have highlighted cephalometric parameters, polysomnographic results [16], the accuracy between surgical planning and achieved post-operative results [17], and clinical results [18].

In order to better understand the correlation between these three parameters and the value of PAS volume, we present a volumetric analysis of PAS changes using a voxel-based three-dimensional evaluation system on CBCT. This analysis involved patients suffering from moderate or severe OSA who underwent CAD/CAM assisted maxillomandibular advancement. 

## 2. Materials and Methods

We performed a retrospective study on patients who underwent MMA because of OSA, operated on consecutively by the same surgeon between 2020 and 2022 at the Maxillo-Facial Surgery and Dental Unit of the Policlinico Hospital in Milan.

According to the Unit’s protocol, all patients had their surgical plans developed using CAD/CAM technology. The inclusion criteria were as follows: (1) moderate or severe OSA confirmed by polysomnography (AHI > 15); (2) multidisciplinary evaluation by the OSA team of our institution (maxillofacial surgeon, pneumologist, ENT surgeon, bariatric surgeon, and dentist), which proposed MMA; (3), drug-induced sleep endoscopy (DISE) performed prior to surgery, confirming the potential benefit of MMA through pull-up mandibular advancement maneuvers; and (4) no anesthesiologic contraindications for performing an elective surgery (ASA ≤ 3).

We excluded pediatric patients and patients affected by specific craniofacial malformations and respiratory diseases.

For each patient, we collected epidemiological data on age, sex, and pre-operative body mass index (BMI). Polysomnography was performed before (max. 6 months) and 6 months after surgery by our Pneumology Unit.

**Figure 1:**
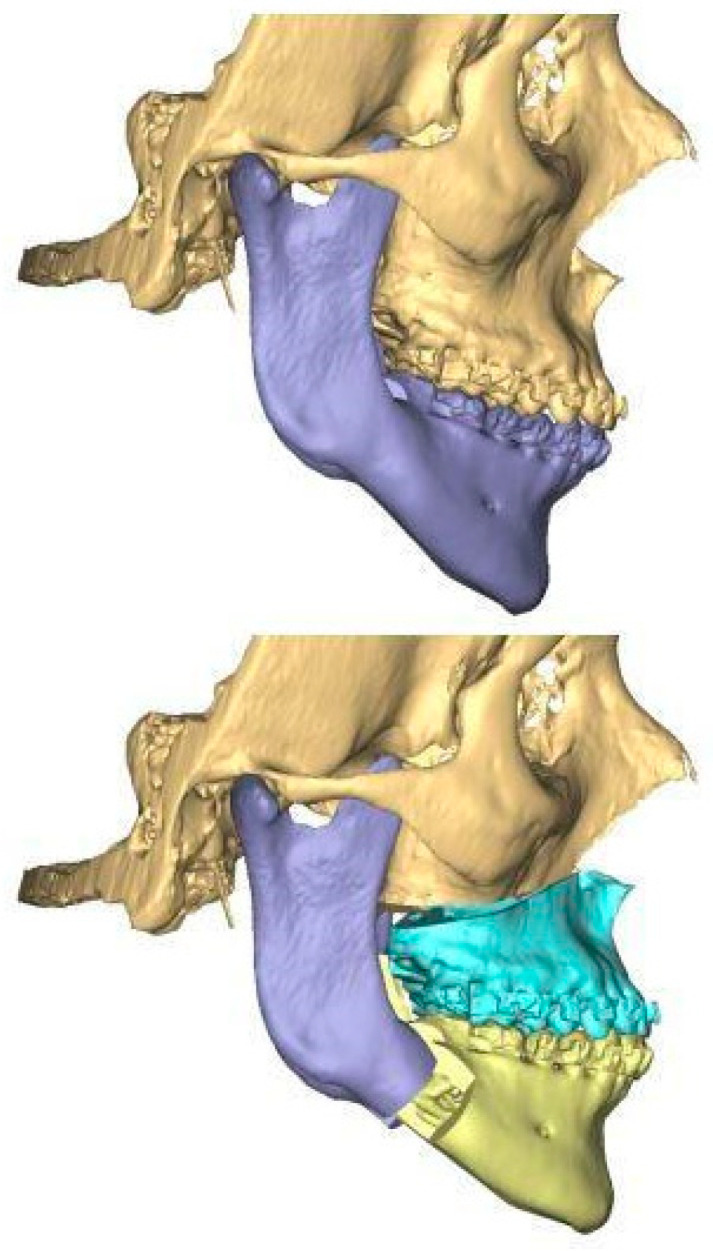
Virtual simulation of MMA (patient N°4).

As with all orthognathic patients undergoing virtual surgical planning with PSI (patient-specific implant) production (e.g., cutting guide and fixation plate), CBCT images of the maxillofacial bones were collected before and 6 months after surgery. All patients benefitted from pre-operative orthodontic treatment in order to improve the occlusal results, and digital intra-oral scans were obtained at the end of this treatment. The STL and DICOM files were merged in order to plan the virtual surgery using the web based application PROPLAN CMF Online 4.0 (DePuy/Synthes (Synthes Inc., 1302 Wrights Lane East, West Chester, PA, USA) in cooperation with Materialise NV, Technologielaan 15 B-3001 Leuven, Belgium) (Figure 1).

**Figure 2:**
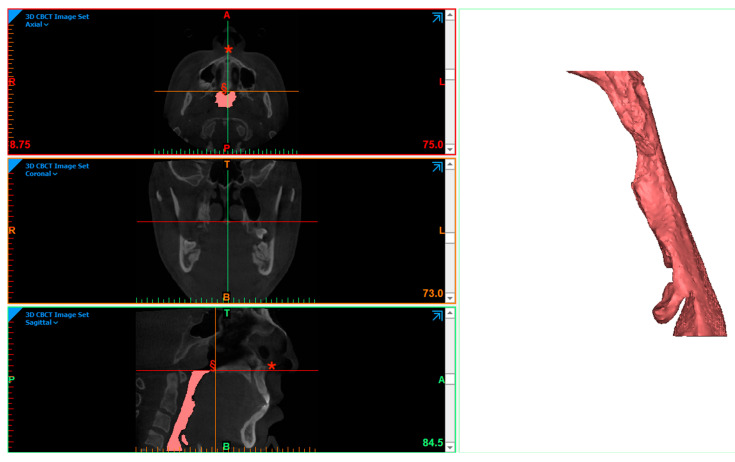
PAS segmentation session on CBCT; *: ANS; §: PNS (patient N°4).

The evaluations of the posterior airway space of the oropharynx were performed using Mimics software (Materialise, Leuven, Belgium) by a single operator. The area of interest (airway space) was identified through manual thresholding with a range from −1024 to −650 Hounsfield Units (HU). Segmentation of the PAS was then performed by selecting, splitting, and cropping a mask on the mid-sagittal slices for the best alignment of the anterior nasal spine (ANS) and posterior nasal spine (PNS). The horizontal plane passing through the PNS was considered the superior border. The inferior border was a horizontal line in the mid-sagittal slice passing through the most inferior point of the hyoid bone. The lateral and posterior boundaries were represented by the pharyngeal walls and the anterior boundary, the soft palate, and the base of the tongue. The “Part” command allowed us to obtain the 3D object of the PAS (Figure 2), and the software automatically calculated the volume of interest in mm^3^ and the min-CSA in mm^2^.

For descriptive statistics, the patients’ data were summarized using mean values and standard deviations for normally distributed quantitative variables. For non-Gaussian variables, median values and 95% confidence intervals (CI) were used. The D’Agostino and Pearson omnibus test was used to assess the normality of the distributions. Measurements taken before and after surgery of the variables considered were compared using the paired Student’s *t*-test (for normally distributed data) or the Wilcoxon matched-pairs signed-rank test (for non-normally distributed data). A significance level of *p* = 0.05 was used. Statistical analysis was performed with GraphPad version 5.03 software.

## 3. Results

Ten patients (nine men and one woman), with a mean age of 51 years and a mean BMI of 27 kg/m^2^ (Table 1), were included in this study.

All subjects underwent bimaxillary osteotomy surgery under general anesthesia, starting with mandibular sagittal osteotomy using PSI, including cutting guides and custom-made mandibular plates, followed by maxillary Lefort I osteotomy performed according to the traditional technique. Final occlusion was ensured using a resin interdental splint device anchored to the teeth with steel wires. One patient needed simultaneous genioplasty. No intra- or post-operative complications occurred. All patients were discharged on the third post-operative day, after 48 h of antibiotic prophylaxis, with InterMaxillary Fixation (IMF) with elastics.

The mean pre-surgical AHI was 52.31 events/h, ranging from 24.5 to 95 (SD 19.02), while the mean post-surgical AHI was 5.94 events/h (SD 5.34) (Table 1).

The AHI showed a statistically significant improvement (Wilcoxon matched-pairs signed-rank test, *p* value 0.004).

The mean pogonion advancement was 14.5 mm, ranging from 13.8 to 15.6 (Table 2).

The mean pre-surgical PAS volume was 8933 mm^3^, and the post-surgical volume was 10,609 mm^3^. In eight out of ten patients, the volume increased with a mean increase of 2640 mm^3^ (max. 5183, min. 951), corresponding to a mean variation of +37.5%. In two patients, the volume decreased by 1591 (−16%) and 2767 mm^3^ (−31%), respectively. The overall mean variation was 25.35%.

The difference between pre- and post-operative results was not statistically significant (paired *t*-test, *p* value 0.033).

The mean min-CSA was 111 mm^2^ pre-surgery and 140 mm^2^ post-surgery. For the eight patients for whom the value increased, the mean increase was 41 mm^2^. For patients n°2 and n°5, the min-CSA decreased by 17 and 27 mm^2^, respectively.

The difference between pre- and post-operative results was not statistically significant (paired *t*-test *p* value 0.146) (Table 2).

## 4. Discussion

OSA is a complex pathology necessitating a multidisciplinary team to ensure a proper treatment plan tailored to the patient’s specific pathological and personal circumstances. The keystone of treatment is to avoid upper airway obstruction during sleep and ensure proper airflow [19]. This goal can be achieved by modifying the airflow intensity (sleeping ventilation with c-PAP), improving muscle tone (hypoglossal neurostimulation), or changing the morphology of the upper airway, generally by increasing its volume.

Among the different methods to change the PAS morphology, orthognathic surgery, including MMA, has become an effective, safe, and long-term stable option [20,21,22]. The effectiveness of MMA is well-known and has been widely investigated according to different parameters, such as aesthetics, polysomnographic data, cephalometric analysis, and patient satisfaction [23,24,25]. Furthermore, the effectiveness of this surgical technique has improved thanks to virtual surgical planning and 3D printing of patient-specific cutting guides and fixation plates [26]. CAD/CAM technology has been evaluated in terms of reducing surgical planning time [27] and in terms of accuracy and predictability [28,29].

The correlation between MMA, PAS volume increase, and AHI reduction has already been described [30,31,32,33], but the ratio between these parameters has yet to be assessed in vivo in patients affected by OSA [34]. According to a recent study on cadavers by Patel et al., the greatest modifications in airway volume are obtained with 6–8 mm of MMA. Today, it is commonly accepted to advance the pogonion by at least 1 cm in eumorphic patients, and as much as possible in patients with dentoskeletal anomalies, to maximize PAS volume—the parameter that must be improved in order to reduce AHI. Unfortunately, it can be harder to obtain sagittal movements with antero-posterior advancement. A study published in 2016 by Baan et al. on ten patients who underwent to CAD/CAM orthognathic surgery supports the idea that, unlike left–right movements, which showed good correspondence between planning and reality, sagittal movements were less accurate, for both the maxilla and mandible [35]. In MMA for OSA, sagittal movements are fundamental, as they are associated with counterclockwise rotation. This pitch movement is frequently needed to avoid excessive anesthetic bimaxillary protrusion, but it increases the risk of relapse due to greater maxillary instability [36]. Both movements are supposed to increase PAS volume, a secondary parameter that has become relevant in assessing the benefits of the procedure, alongside AHI reduction [15,37].

Very interestingly, for the first time in the literature, we present two patients (N°2 and N°5) who do not follow this rule: even with the 6-month AHI showing a significant reduction, their PAS volume decreased. Unlike the other patients, these two patients presented with asymmetrical occlusion, so the virtual planning consisted of rotational movements and not only advancement. We imagine that the rotational movements balanced the advancements due to soft tissue modifications. This finding supports the idea that the extent of some orthognathic movements could be reduced, thus avoiding an unstable maxillomandibular position. Despite these volumetric results, the effectiveness in OSA treatment was excellent, with a, AHI of 3.8 for both patients at 6 months post-surgery. Interestingly, in our series, the two patients with the highest PAS volume increases—N°1 and N°7 (+5057 mm^3^ [+60.5%] and +5182 mm^3^ [61%] respectively)— also correspond to the lowest pogonion advancements (13.8 mm).

The reported “anomalies” should be considered confirmation of the extremely complex morpho-functional anatomy of OSA, in which the tone and dynamics of pharyngeal and tongue muscles certainly play an important role [38]. Moreover, we have been able to find some articles in the literature that suggest different points of view on MMA, PAS modifications, and AHI reduction. Zhou et al. published a study in 2022 analyzing 100 patients who underwent MMA for moderate to severe OSA, reporting a success rate of 67%. Their analysis suggests that pre-existing CVD, a larger PAS, and a higher central apnea index are correlated with less favorable outcomes after MMA. This study confirms that PAS is not a universal parameter for evaluating MMA effectiveness [39]. The downsizing of the role of PAS could also be supported if we consider that setback mandibular movement, with PAS decrease, does not lead to OSA [40]. The complexity of PAS volume and OSA is also shown by our post-operative modifications not being statistically significant.

Our study confirms that MMA is very effective for OSA, with seven patients cured (post-operative AHI < 5) and three patients showing a substantial AHI reduction of less than 50% of their preoperative values. In the literature, it is always presented that this AHI result corresponds with PAS volume increase, but we have shown that this is not true and that the correlation is not as linear as expected, Despite the littlest PAS volume (3781 mm^3^) being measured in the patient with the highest AHI value (95/h), we obtained very variable results in AHI, even among patients with similar PAS volumes. Moreover, small percentage increases in PAS volume resulted in significant AHI decreases (e.g., patients N°4 and N°9 with volume increases of 6 and 10%, respectively, and both showing a non-pathological post-operative AHI of 2.4 and 4.6). The effectiveness of MMA with AHI reduction, even without PAS volume improvement, suggests that the benefits of MMA likely stem from increased muscle tone and stiffness, which is not reflected in airway volume, but in a reduction in soft tissue collapse. More morphofunctional evaluations are needed to better explain OSA pathophysiology and obtain a perfectly tailored treatment for each patient. A volumetric and morphometric analysis of lingual and pharyngeal muscles using pre- and post-operative MR, would surely be interesting to better understand how MMA could heal OSA. Another field of research would surely be the correlation and predictability of PAS volume modifications based on the extent of maxillomandibular movements, which should be planned not only for PAS volume increase but also considering their effects on muscles variations.

## 5. Conclusions

MMA, virtually planned with PSI, is confirmed as an effective treatment for OSA, reducing AHI. However, volumetric analysis of PAS shows intriguing results, such as PAS volume reduction despite AHI improvement. This suggests that more evaluations, including more data on muscle modifications, need to be performed.

## Figures and Tables

**Table 1:** Characteristics of the patients under study.

Patient	Gender	Age	BMI (kg/m^2^)	AHI pre	AHI Post
1	M	54	25.7	65	3.5
2	M	50	29.6	33.5	3.8
3	M	46	35	58	4.1
4	M	61	25.9	54	2.4
5	M	30	22.1	39.4	3.8
6	M	50	33.3	52.5	4.4
7	M	54	21	50.2	2.6
8	M	48	22.2	51	9
9	F	60	22.3	24.5	4.6
10	M	57	32.8	95	20.2

**Table 2:** Degree of pogonion advancement (mm), and pre- and post-operative min-CSA (mm^2^) and volumetric results of PAS (mm^3^) with percentage.

Patient	Pog Adv	Pre Min CSA	Post-Min CSA	Change	Pre Volume	Post Volume	Change	% Variation
1	13.8	73.708	260.8	187.092	8353.37	13,411.32	5057.95	60.5%
2	14.7	170.847	153.756	−17.091	9896.26	8304.74	−1591.559	−16%
3	14.9	57.179	88.633	31.454	4764.63	6183.19	1418.566	29%
4	14.5	145.77	222.343	76.573	14,877.69	16,496.57	1618.88	10%
5	14.7	128.042	101.226	−26.816	8686.75	5919.04	−2767.714	−31%
6	15.2	73.708	77.92	4.212	9148.74	10,637.3	1488.559	16%
7	13.8	167.22	168.34	1.12	8488.37	13,671.32	5182.95	61%
8	14.1	57.179	77.54	20.361	6074.04	8559.76	2485.714	40%
9	14.0	117.56	129.05	11.49	15,268.69	16,219.57	950.88	6%
10	15.6	122.15	124.75	2.599	3781.63	6698.19	2916.566	78%

## Data Availability

Dataset available on request from the authors.
